# Metastasis-associated in colon cancer-1 promotes vasculogenic mimicry in gastric cancer by upregulating TWIST1/2

**DOI:** 10.18632/oncotarget.3416

**Published:** 2015-03-20

**Authors:** Lin Wang, Li Lin, Xi Chen, Li Sun, Yulin Liao, Na Huang, Wangjun Liao

**Affiliations:** ^1^ Department of Oncology, Nanfang Hospital, Southern Medical University, Guangzhou, China; ^2^ Department of Cardiology, Nanfang Hospital, Southern Medical University, Guangzhou, China

**Keywords:** metastasis-associated in colon cancer-1, TWIST1/2, vasculogenic mimicry, gastric cancer

## Abstract

Vasculogenic mimicry (VM) is a blood supply modality that is strongly associated with the epithelial-mesenchymal transition (EMT), TWIST1 activation and tumor progression. We previously reported that metastasis-associated in colon cancer-1 (MACC1) induced the EMT and was associated with a poor prognosis of patients with gastric cancer (GC), but it remains unknown whether MACC1 promotes VM and regulates the TWIST signaling pathway in GC. In this study, we investigated MACC1 expression and VM by immunohistochemistry in 88 patients with stage IV GC, and also investigated the role of TWIST1 and TWIST2 in MACC1-induced VM by using nude mice with GC xenografts and GC cell lines. We found that the VM density was significantly increased in the tumors of patients who died of GC and was positively correlated with MACC1 immunoreactivity (*p* < 0.05). The 3-year survival rate was only 8.6% in patients whose tumors showed double positive staining for MACC1 and VM, whereas it was 41.7% in patients whose tumors were negative for both MACC1 and VM. Moreover, nuclear expression of MACC1, TWIST1, and TWIST2 was upregulated in GC tissues compared with matched adjacent non-tumorous tissues (*p* < 0.05). Overexpression of MACC1 increased TWIST1/2 expression and induced typical VM in the GC xenografts of nude mice and in GC cell lines. MACC1 enhanced TWIST1/2 promoter activity and facilitated VM, while silencing of TWIST1 or TWIST2 inhibited VM. Hepatocyte growth factor (HGF) increased the nuclear translocation of MACC1, TWIST1, and TWIST2, while a c-Met inhibitor reduced these effects. These findings indicate that MACC1 promotes VM in GC by regulating the HGF/c-Met-TWIST1/2 signaling pathway, which means that MACC1 and this pathway are potential new therapeutic targets for GC.

## INTRODUCTION

Cancer requires an adequate blood supply to sustain rapid growth [1]. It was long believed that only endothelial cells could form blood vessels. However, when endothelium-dependent vessel growth is insufficient to support the rapid proliferation of tumor tissues, nonendothelial vascular networks also originate from tumors through a process called vasculogenic mimicry (VM) [2]. VM is the dominant method that provides the blood supply in the early stage of cancer and is also an important route of metastasis [3]. It has been demonstrated that patients with tumor-associated VM have a shorter survival time and a higher rate of metastasis than patients without it [4–6]. In addition, VM is likely to limit the effectiveness of anti-angiogenesis agents [7]. Although ramucirumab, a monoclonal antibody vascular endothelial growth factor receptor (VEGFR)-2 antagonist, in combination with paclitaxel would increase overall survival in patients treated for advanced gastric cancer (GC) [8], drugs such as bevacizumab that target vascular endothelial growth factor (VEGF) signaling have failed to prolong the survival of patients with advanced GC in Phase II and III clinical trials [9, 10]. A recent study showed that VM was still markedly increased in the tumors of patients receiving anti-angiogenesis treatment for ovarian carcinoma [11], indicating that VM is a potential alternative for providing the blood supply to maintain tumor growth when endothelium-dependent angiogenesis is inhibited. Many authors have conducted investigations of VM in a variety of tumors [2, 4, 6, 11, 12], but only a few studies have focused on GC [5, 13]. It was reported that VM occurs in poorly differentiated human GC [5] and that the anti-VM effect of the IRX1 tumor suppressor gene contributes to inhibition of metastasis in animals with GC xenografts [13]. However, evidence is limited with regard to whether VM has any influence on the prognosis of GC patients and the underlying mechanisms remain unclear.

We previously reported that upregulation of metastasis-associated in colon cancer-1 (MACC1) predicts a poor clinical outcome for patients with GC, and that MACC1 promotes GC cell proliferation and invasion by inducing the epithelial-mesenchymal transition (EMT) through activation of the hepatocyte growth factor (HGF)/c-Met signaling pathway [14]. In addition, HGF induces nuclear translocation of MACC1 that is required for its biological activity [15], while the EMT is known to play a pivotal role in tumorigenesis [16, 17] and in VM [18, 19]. Therefore, we hypothesized that MACC1 has a role in the process of VM in GC.

TWIST1 potently induces the EMT [20–22] and also has a critical role in the occurrence of VM in hepatocellular carcinoma [23]. TWIST2 is a member of the basic helix-loop-helix (bHLH) transcription factor family, and it shares more than 90% sequence homology and structural similarity with TWIST1 at the bHLH and C-terminal domains [24]. The ability of TWIST2 to mediate the EMT in several types of cancer [25, 26] suggests that it may also be active in VM, but a definite role for TWIST2 in the VM process has not been reported. We hypothesized that nuclear translocation of MACC1 promotes VM in GC by activating the TWIST1/2 signaling pathway.

In this study, we first examined whether MACC1 was associated with VM in GC and whether detection of VM predicted the prognosis of 88 GC patients. Then we investigated the role of MACC1 in the occurrence of VM in nude mice with GC xenografts and in cultured GC cell lines. Finally, we determined whether the HGF/c-Met-MACC1-TWIST1/2 signaling pathway was the underlying mechanism of VM.

## RESULTS

### VM in GC tissues predicts an adverse clinical outcome

**Figure 1:**
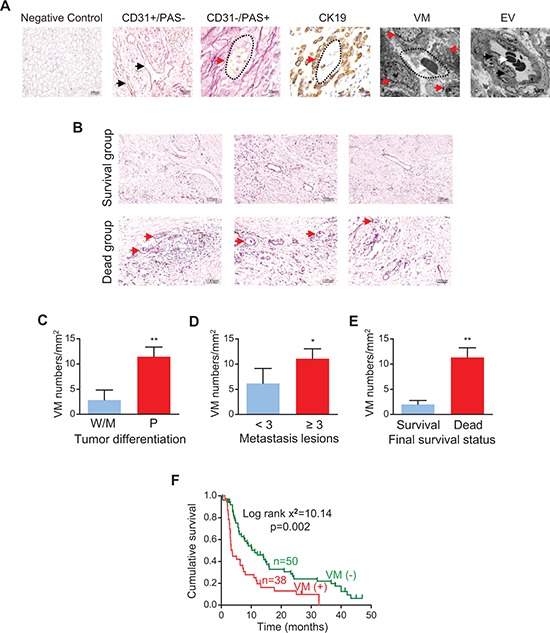
VM in human gastric cancer (GC) tissues and its relation with the clinical outcome **(A)** Representative pictures of vessels formed in GC tissues by endothelium-dependent angiogenesis and structures formed by vasculogenic mimicry (VM), which were confirmed by CD31/PAS staining and transmission electron microscopy (TEM). Negative control is indicated in panel 1. CD31-positive structures (brown color, indicated by black arrows in panel 2) are vessels with an endothelial lining, while PAS-positive networks are VM channels lined by tumor cells (pink color, indicated by red arrow in panel 3). CK19-positive staining shows VM is surrounded by tumor cells (brown color, indicated by red arrows in panel 4). Panels 5 and 6 show VM structure (red arrows) and endothelial cells of a blood vessel (black arrows) by TEM, respectively; several blood cells (white arrows) are surrounded by a complete vessel wall. Original magnification: × 200 (panel 1); × 400 (panel 2); × 1000 (panels 3 and 4); × 8000 (panel 5); × 5000 (panel 6). **(B)** Representative photomicrographs showing VM density in survivors and non-survivors. **(C–E)** VM density in GC patients stratified by tumor differentiation [W, well differentiated; M, moderately differentiated; P, poorly differentiated (C)], metastasis (D), and survival (E). *p < 0.05; ^*^*p < 0.01 versus the corresponding control group, *n* = 14 and *n* = 74 for the W/M group and P group, respectively; *n* = 18 and *n* = 70 for < 3 and ≥ 3 metastasis lesions, respectively; *n* = 12 and *n* = 76 for survivors and non-survivors, respectively. **(F)** Kaplan-Meier plot showing the influence of VM density on the survival of patients with stage IV GC.

To confirm whether VM could be detected in tumor tissues obtained from 88 patients with stage IV GC, we used staining for platelet endothelial cell adhesion molecule-1 (CD31) to identify the endothelium in GC tissue sections, as well as periodic acid-Schiff (PAS) stain to identify extracellular matrix-rich channels between tumor cells. Accordingly, CD31 positive vessels were derived from endothelial cells (Figure 1A, 2nd panel), while PAS positive channels represented VM (Figure 1A, 3rd panel). Labeling for cytokeratin 19 (CK19) demonstrated VM structures were formed by dense tumor cells (Figure 1A, 4th panel). Hematoxylin and Eosin (H&E) staining showed red blood cells in VM structures (Figure S1). Transmission electron microscopy showed that tumor vessels arising from conventional angiogenesis contained erythrocytes and were lined by flattened endothelial cells (Figure 1A, 6th panel), while channels created by VM were surrounded by tumor cells (Figure 1A, 5th panel) and were structurally consistent with “VM” as defined by Maniotis and colleagues [2]. We found VM in 43.0% of the GC patients (38/88 tumor samples).

We further assessed the relationship between VM density and clinicopathological or prognostic parameters. The VM density was higher in non-survivors than in survivors (Figure 1B). VM density was positively correlated with tumor differentiation (*p* < 0.001), metastasis (*p* = 0.017) and survival status (*p* < 0.001, Figure 1C to 1E). Kaplan-Meier analysis showed that patients with a higher VM density in tumor tissue had a lower overall survival rate (*p* = 0.002, Figure 1F). Univariate and multivariate analyses both demonstrated that VM was an important prognostic factor for patients with GC (*p* = 0.005, Table S1 and S2). Taken together, these findings indicate that VM predicts a worse outcome in patients with advanced GC.

### MACC1 expression is correlated with VM density in GC

**Figure 2:**
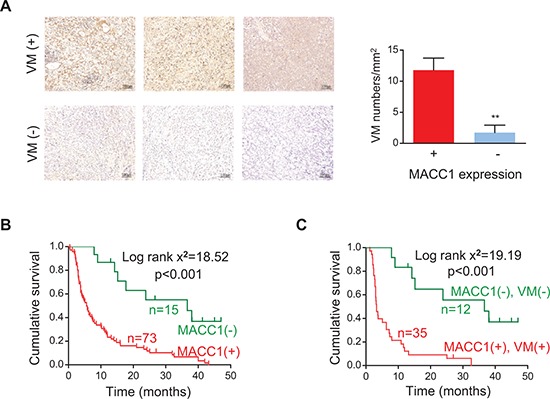
Relation between MACC1 expression and VM density in human GC tissues and their combined influence on survival **(A)** Immunostaining for MACC1 in GC tissues with higher and lower VM density. **p < 0.01, *n* = 73 and *n* = 15 for MACC1-positive and MACC1-negative tumors, respectively. **(B, C)** Kaplan-Meier survival analysis of GC patients categorized by MACC1 expression (B) and by a combination of MACC1 expression and VM density (C).

Since we have previously demonstrated that upregulation of MACC1 predicts a poor prognosis of GC [14], we examined the correlation between MACC1 expression and VM density in the present study. We found that MACC1 expression was upregulated in the higher VM density group compared with the lower VM density group (Figure 2A). MACC1 expression was positively correlated with VM density (*r* = 0.212, *p* = 0.047) and with the expression of vascular endothelial cadherin (VE-cadherin), an important regulator of VM (*r* = 0.487, *p* < 0.001, Table S3). Kaplan-Meier analysis revealed that patients with high levels of MACC1 expression in their tumors had a significantly lower overall survival rate (Figure 2B). Interestingly, the 3-year survival rate was only 8.6% for patients whose tumors showed double positive staining for MACC1 and VM, whereas it was 41.7% for patients with tumors that were negative for both MACC1 and VM. The median survival time was 3.3 versus 36.0 months, respectively (Figure 2C). These results suggested that MACC1 is associated with VM in GC, but the mechanism involved was unknown.

### MACC1 promotes VM both *in vivo* and *in vitro*

**Figure 3:**
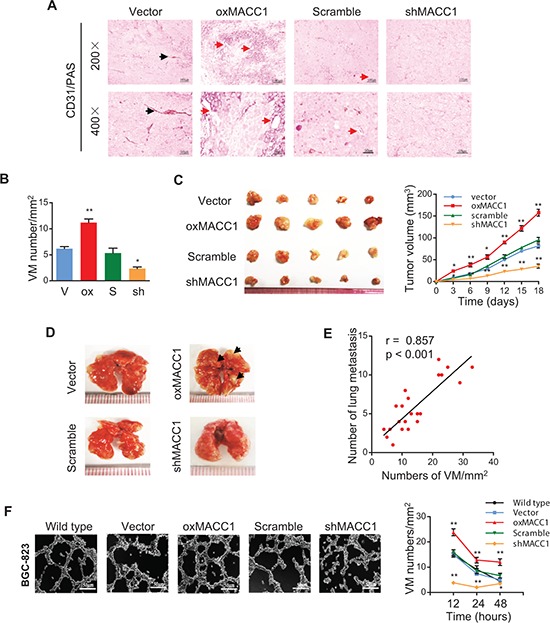
MACC1 promotes VM *in vivo* and *in vitro* **(A)** Representative CD31/PAS stained images of tumor sections from GC xenografts with overexpression or silencing of MACC1 (oxMACC1 and shMACC1) and their corresponding controls. Red arrows indicate a typical VM structure. Black arrows indicate a endothelial vessel. **(B)** Quantitation of VM density in GC xenografts shows that it is higher in the MACC1-overexpressing group than in other groups. *p < 0.05; **p < 0.01, *n* = 6 vs. the corresponding control group. **(C)** Size (left panel) and tumor volume curves (right panel) of GC xenografts harvested at 18 days after inoculation. *p < 0.05; **p < 0.01, *n* = 6. **(D)** Metastases (black arrows) in the lungs at 40 days after inoculation. Metastases were frequent in the MACC1-overexpressing group, but were seldom detected in the MACC1-silenced group. **(E)** The VM density of xenograft GC tissues was positively correlated with the number of lung metastases (*r* = 0.857, *p* < 0.001; *n* = 6). **(F)** Representative VM images and quantitation of tube formation by BGC-823 GC cells after 3D culture for 12 hours. Scale bar = 50 μm. *p < 0.05; **p < 0.01, *n* = 3.

To investigate whether MACC1 contributes to VM, we generated subcutaneous GC implantation and lung metastasis models in NOD-SCID nude mice (*n* = 6/group), as described previously [14]. We also established BGC-823 cell lines with stable overexpression of the MACC1 gene (oxMACC1) and with silencing of MACC1 (shMACC1). As shown in Figure 3A and 3B, CD31/PAS staining revealed that VM was significantly increased in oxMACC1 GC xenografts compared with the vector-control group (*p* = 0.002). In contrast, VM was markedly reduced in shMACC1 xenografts compared with the scramble-control group (*p* = 0.012). Tumors were larger and there were more lung metastases in the oxMACC1 group than in the vector-control group, while shMACC1 tumors were significantly smaller and pulmonary metastases were fewer than in the scramble-control group (Figure 3C and 3D). Strikingly, the VM density in xenograft GC tissues was strongly correlated with the number of lung metastases (*r* = 0.857, *p* < 0.001, Figure 3E), suggesting that VM is associated with metastasis of GC.

Based on these findings, we used a well-established three-dimensional (3D) culture model with poorly-differentiated BGC-823 and well-differentiated MKN28 cells to investigate the effect of MACC1 on VM. In 3D culture, oxMACC1 BGC-823 cells showed dramatic reorganization and formed typical tube-like structures, with the number of tubes reaching a peak at 12 hours (*p* = 0.005, Figure 3F), while the peak of VM network formation was seen at 48 hours in cultures of MKN-28 cells (*p* < 0.001, Figure S2). In contrast, tube formation was not obvious in 3D cultures of shMACC1 cells. These results indicated that MACC1 promotes both VM and tumor progression.

### MACC1 upregulates TWIST1/2 in GC

**Figure 4:**
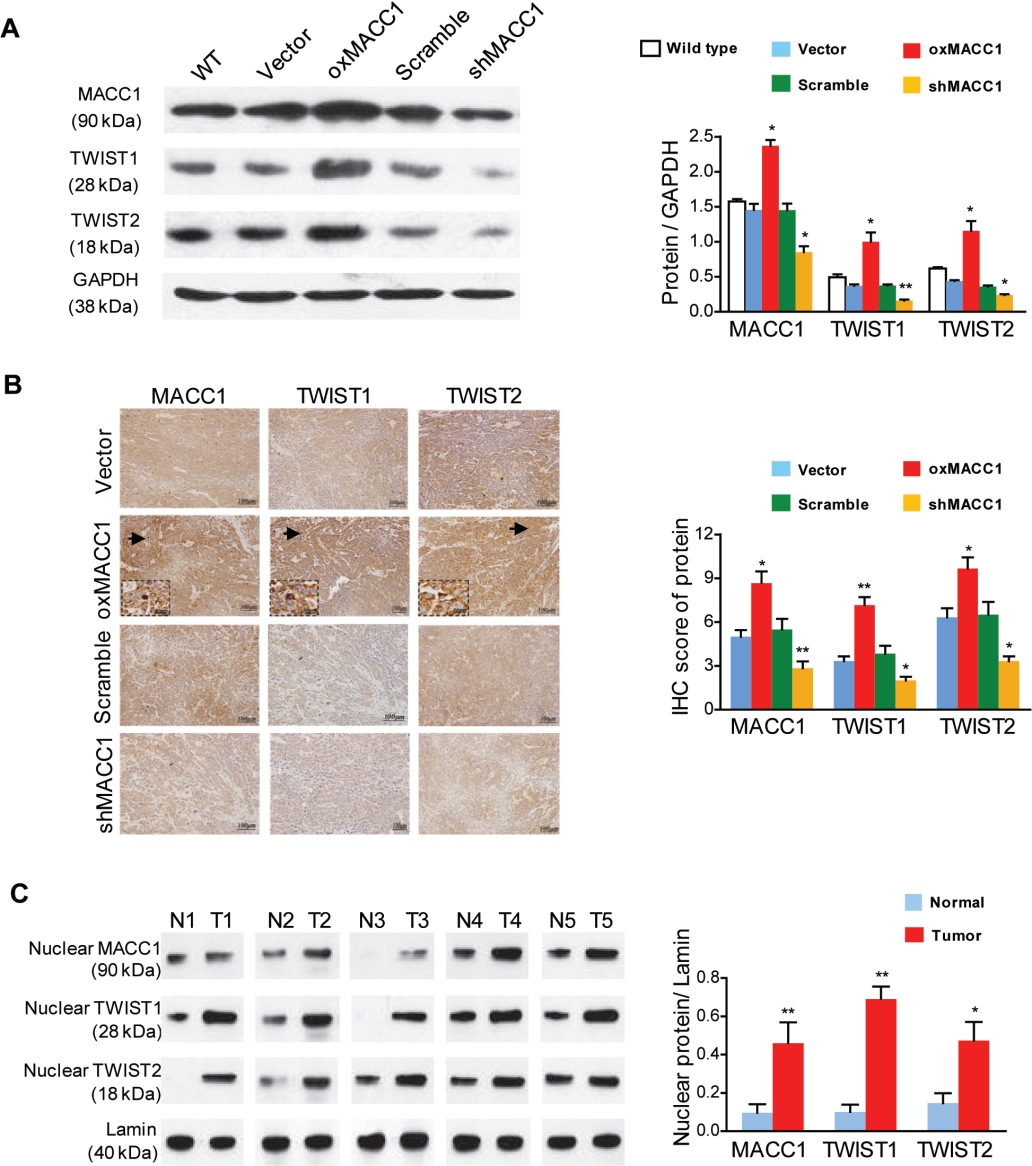
MACC1 upregulates TWIST1 and TWIST2 expression in GC cells and tumor tissues **(A)** Western blot analysis (left panel) and quantitation (right panel) of MACC1, TWIST1, and TWIST2 expression in response to overexpression or silencing of MACC1 (oxMACC1 and shMACC1) in BGC-823 cells. *p < 0.05; **p < 0.01, *n* = 3. WT, wild type. GAPDH was used as a loading control. **(B)** Representative images (left panel) and quantitation (right panel) of MACC1, TWIST1, and TWIST2 immunostaining in xenograft GC tissues. *p < 0.05; **p < 0.01, *n* = 6. **(C)** Nuclear expression of MACC1, TWIST1 and TWIST2 proteins in human primary GC tissues (T) and in matched adjacent non-tumor tissues (N) Lamin was used as the loading control. *p < 0.05; **p < 0.01, *n* = 5.

Previous reports have indicated that expression of TWIST1, a key regulator of the EMT [22], is biologically and clinically linked to VM in tumors [18, 23]. Although TWIST2 has a similar sequence to TWIST1 [24], its role in the process of VM is unknown. We investigated whether TWIST1/2 were involved in MACC1-induced VM in GC. As shown in Figure 4A and Figure S3, the expression of TWIST1 and TWIST2 proteins was upregulated in oxMACC1 GC cell lines, but was downregulated in shMACC1 GC cell lines. In GC xenograft nude mice, immunohistochemistry showed that expression of TWIST1 and TWIST2 was significantly increased in the oxMACC1 group and was decreased in the shMACC1 group (*p* = 0.004 and *p* = 0.022, Figure 4B). In addition, we found that the nuclear expression of MACC1, TWIST1, and TWIST2 proteins was significantly higher in the tumor tissues of VM-positive GC patients than in their matched adjacent non-tumor tissues (*p* = 0.045, *p* = 0.015, and *p* = 0.007, Figure 4C).

In order to determine whether the upregulation of TWIST1/2 by MACC1 generally exists in the carcinogenesis and tumor progression of multiple cancers, we chose three poorly differentiated cancer cell lines, including human colon cancer cell lines (HCT-116 and SW480), breast cancer cell lines (MDA-MB-231 and BT549) and lung cancer cell lines (A549 and NCIH520). We found that expression of MACC1 was greatly higher in HCT-116, BT549 and NCIH520 cell lines than in SW480, MDA-MB-231 and A549 cell lines (*p* = 0.002, *p* = 0.032 and *p* = 0.003, Figure S4A). By knocking down the MACC1 gene, expression of TWIST1 and TWIST2 was also significantly downregulated in HCT-116, BT549 and NCIH520 cell lines (*p* < 0.05, Figure S4B). These findings suggest that MACC1 may promote VM by upregulating TWIST1/2 in multiple cancers.

### MACC1 increases transcriptional activity of TWIST1/2

**Figure 5:**
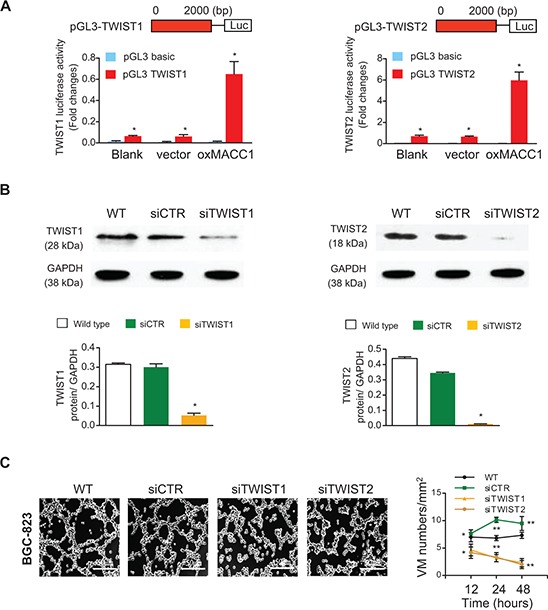
MACC1 increases TWIST1/2 transcriptional activity in BGC-823 cells **(A)** Luciferase activity assays in 293T cells showed the enhancement TWIST1 and TWIST2 promoter activity by MACC1. **(B)** TWIST1 and TWIST2 proteins were silenced by the corresponding siRNA sequences. **(C)** Representative images of VM density (tube formation) after 24 hours of treatment and the time course of VM density in 3D-cultures of BGC-823 cells treated with silenced TWIST1 or TWIST2, or with the siCTR control. *p < 0.05; **p < 0.01, *n* = 3 in each group.

We further investigated how MACC1 affected the expression of TWIST1 and TWIST2. The luciferase reporter assay showed that luciferase activity driven by the TWIST1 and TWIST2 promoter was increased in the oxMACC1 group compared with the vector-control group (*p* = 0.036 and *p* = 0.017, Figure 5A), suggesting that MACC1 transcriptionally upregulates TWIST1 and TWIST2. Importantly, we found that silencing of TWIST1 and TWIST2 significantly reduced tube formation by GC cells, indicating that these molecules play a central role in MACC1-induced VM in GC (Figure 5B and 5C, Figure S5A and S5B).

### HGF/c-Met signaling is required for MACC1-induced VM

**Figure 6:**
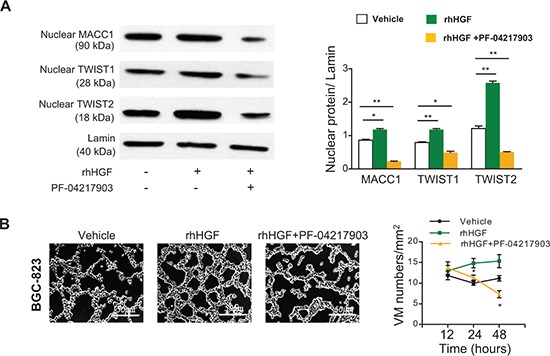
HGF/c-Met contributes to MACC1-induced VM in BGC-823 cells **(A)** Nuclear expression of TWIST1 and TWIST2 was upregulated by recombinant human hepatocyte growth factor (rhHGF), while this change was antagonized by co-treatment with a c-Met inhibitor (PF-04217903). *p < 0.05; **p < 0.01, *n* = 3 in each group. **(B)** Effect of rhHGF and PF-04217903 on VM. Representative pictures were taken after 24 hours of treatment with the indicated agents. Scale bar = 50 μm. Effect of rhHGF, rhHGF+PF-04217903, or the vehicle on the VM density over time. *p < 0.05; **p < 0.01, *n* = 3 in each group. The concentrations of rhHGF and PF-04217903 were 20 μM and 22 μM, respectively.

It has been reported that HGF/c-Met activation leads to nuclear translocation of MACC1 [15], and that c-Met promotes the process of VM [27], implying that the HGF/c-Met pathway might be involved in MACC1-induced VM. To clarify this issue, we treated GC cells with recombinant human HGF (rhHGF) and a c-Met inhibitor (PF-04217903). We found that rhHGF stimulated GC cell proliferation in a dose-dependent manner. As shown in Figure S6A, 20 μM of rhHGF significantly increased the proliferation of BGC-823 cells by 25% and MKN-28 cells by 15% compared with their corresponding controls at 24–48 hours. In addition, the half maximal inhibitory concentration (IC_50_) of PF-04217903 was 22 μM for BGC-823 cells and 2 μM for MKN-28 cells at 24–48 hours. We chose 20 μM of rhHGF and 22 μM/2 μM of c-Met inhibitor as the concentrations for subsequent experiments using BGC-823 and MKN-28 cells. Western blotting showed that the nuclear expression of MACC1, TWIST1, and TWIST2 proteins was increased in cells treated with rhHGF, while this increase was abolished by co-treatment with the c-Met inhibitor PF-04217903 (Figure 6A and Figure S6B). In addition, the number of tubes formed by VM in 3D cell culture was increased by rhHGF, while co-treatment with PF-04217903 antagonized this effect (Figure 6B and Figure S6C).

### MACC1 upregulates key downstream effectors of the TWIST1/2 signaling pathway

**Figure 7:**
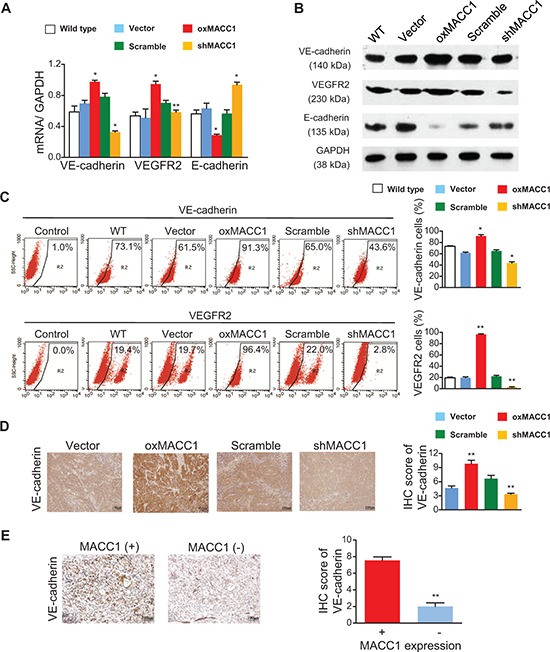
Effect of MACC1 on expression of VE-cadherin, VEGFR2, and E-cadherin Overexpression of MACC1 (oxMACC1) or silencing of MACC1 (shMACC1) altered the expression of VE-cadherin, VEGFR2, and E-cadherin mRNA **(A)** and protein **(B)** in BGC-823 cells. **(C)** VE-cadherin and VEGFR2 expression in BGC-823 cells were assessed by flow cytometry. *p < 0.05; **p < 0.01, *n* = 3 in each group. **(D)** VE-cadherin expression was increased in oxMACC1 xenograft GC tissues and decreased in shMACC1 tissues, **p < 0.01, *n* = 6 in each group. **(E)** VE-cadherin expression in human GC tissues with positive or negative immunostaining for MACC1. **p < 0.01, *n* = 73 and *n* = 15 for MACC1 positive or MACC1 negative, respectively.

VE-cadherin [28] and vascular endothelial growth factor receptor 2 (VEGFR2) [18, 29] are key downstream effectors of the TWIST1 signaling pathway, but it was unclear whether these two molecules participate in MACC1-induced VM. As shown in Figure 7A and 7B and Figure S7A and S7B, VE-cadherin and VEGFR2 mRNA and protein expression were markedly increased in oxMACC1 GC cell lines and greatly downregulated in shMACC1 cells. In contrast, E-cadherin expression was strikingly reduced in oxMACC1 cells and was significantly increased in shMACC1 cells. Similar results were obtained using flow cytometry (Figure 7C and Figure S7C). In addition, immunohistochemistry of xenograft GC tissues revealed that VE-cadherin expression was significantly increased in the oxMACC1 group and markedly decreased in the shMACC1 group (*p* = 0.003 and *p* = 0.004, Figure 7D). Furthermore, the expression of VE-cadherin was positively correlated with VM density in the tumor tissues of GC patients (*p* < 0.001, Figure 7E and Table S4). These results suggested that MACC1 induces VM via activation of the “TWIST1/2-VE-cadherin/VEGFR2” axis.

## DISCUSSION

Angiogenesis is known to be essential for tumorigenesis and anti-angiogenesis therapy has become an important approach to the treatment of cancer. Recent studies have shown that development of endothelial-lined vessels is inhibited by some anti-angiogenic agents, but VM and metastasis still increase after anti-angiogenesis therapy [7, 30], which might partly explain the limited efficacy of such treatment. Occurrence of VM at an early stage is thought to be a crucial step in the progression and metastasis of cancers [3–6, 31], suggesting that VM is a potential therapeutic target. Some oncogenes can promote VM [4, 19, 32], but some oncogenes don't exert important role in VM. For example, there is no evidence that the well-known oncogenes K-ras and B-raf influence the VM process [33–35]. Although MACC1 has been demonstrated to play important role in GC, it is completely unknown whether it plays any role in VM process of cancers. In the present study, we provided first evidence that MACC1 expression was correlated with VM density in GC patients, and promoted VM in nude mice with GC xenografts and in cultured GC cell lines. In addition, we firstly clarified that activation of HGF/c-Met-TWIST1/2-VE-cadherin/VEGFR2 signaling pathway contributed to MACC1-induced VM. These findings suggest that inhibition of MACC1 is a potential approach for the treatment of GC, since it would reduce VM by decreasing TWIST1/2 and tumor progression would be slowed.

In the present study, we observed VM in 43.0% of tumor samples from patients with stage IV GC, while it has been reported that 23.0% of GC tissues obtained from patients in stages I–IV show VM [5]. Thus, it is plausible to consider that VM increases as GC progresses. We also found that the overall survival rate was much lower in patients with tumors positive for MACC1 and VM, suggesting that therapy targeting these two molecules might both MACC1 expression and the process of VM be more effective for improving the prognosis.

VM increases the perfusion of rapidly growing tumors by transporting fluid from leaky vessels, and VM tubes may even connect with the endothelial-lined vasculature. Drugs such as bevacizumab, sorafenib, and sunitinib that target endothelial signaling molecules have been used clinically to treat various cancers, including GC, but their efficacy is limited [36], which may be partially attributable to an inability to inhibit VM [37]. It seems reasonable that the most efficient way to target tumor cell plasticity is to inhibit both endothelium-dependent angiogenesis and VM simultaneously. The present study demonstrated that MACC1, HGF/c-Met, TWIST1, and TWIST2 are important proteins facilitating VM, each deserving serious consideration as potential therapeutic targets and diagnostic indicators for aggressive metastatic GC.

The EMT is known to contribute to tumor cell plasticity and is also a characteristic of VM [18]. We previously demonstrated that MACC1 is involved in the EMT [14], supporting our finding in this study that MACC1 promotes VM. TWIST1 is a transcription factor involved in EMT regulation, and it was reported that the Bcl-2/TWIST1 complex facilitates VM in hepatocellular carcinoma [18]. Coincidentally, we found that MACC1 regulates TWIST1 and promotes VM in GC. We also noted that TWIST2 had a similar effect. TWIST1 and TWIST2 share many functions, such as their regulation of hematological malignancies and their role in cancer progression and metastasis [24, 25, 38]. It is known that TWIST1 opens nuclear membrane pores with the help of an accessory protein and enters the nucleus to regulate transcription of downstream genes that are involved in the process of VM [39]. However, there have been no previous investigations into the role of TWIST2 in VM. In the present study, we found that both TWIST1 and TWIST2 were upregulated in oxMACC1 GC cells and were downregulated in shMACC1 cells. Silencing of either TWIST1 or TWIST2 inhibited VM, suggesting that activation of either molecule is sufficient to drive MACC1-induced VM in GC.

It has been reported that MACC1 binds to the promoter of the c-Met gene [15, 40, 41]. Our present findings revealed that HGF/c-Met is not only the downstream link between MACC1 and VM, but also positive feedback stimulation of MACC1 expression, thereby promoting VM. In agreement with our results, previous studies have shown that activation of HGF/c-Met contributes to VM. It was reported that overexpression of c-Met facilitates VM in melanoma [27], while c-Met deletion was associated with the highest rate of TWIST alterations in osteosarcoma [42]. We demonstrated that HGF facilitates nuclear translocation of MACC1 and upregulation of TWIST1/2 to promote VM in GC, whereas a c-Met inhibitor antagonizes this process.

Several key proteins are essential for the occurrence of VM in various cancers [2, 4, 6, 18], among which VE-cadherin is the most critical for vascular signaling cascades. Silencing of VE-cadherin has been reported to result in failure to form a VM network of cord-like structures [28]. E-cadherin is required for adherent junctions, while its deficiency in the cytosolic membrane results in loss of cell polarity and finally facilitates the EMT [43, 44]. Our previous study demonstrated that MACC1 overexpression reduces E-cadherin expression [14], while Sun et al. reported that TWIST1 induces VM in HCC through downregulation of E-cadherin and upregulation of VE-cadherin [18]. In the present study, we showed that the expression of VE-cadherin was positively correlated with the VM density, and that VE-cadherin expression was increased in GC cell lines overexpressing MACC1 while E-cadherin expression was decreased. It is generally agreed that VEGFR-2 is a major mediator of the mitogenic, angiogenic, and permeability-enhancing effects of VEGF [29], which are also involved in VM [18]. The present study showed that VEGFR2 mRNA and protein expression were increased in MACC1 overexpressing GC cells, and were decreased in MACC1 silenced cells.

**Figure 8:**
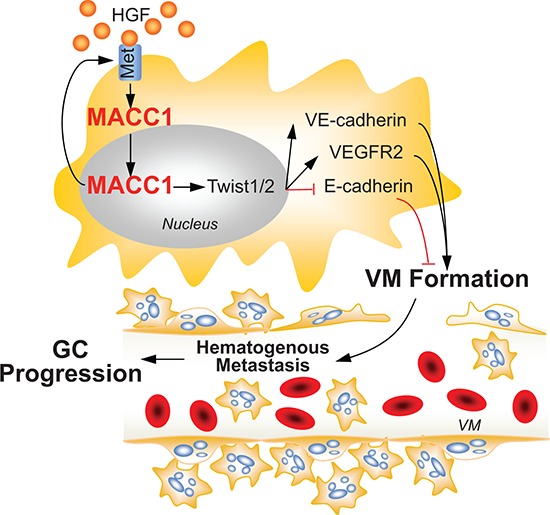
Role of the MACC1-dependent signaling pathway in VM Increased MACC1 expression in gastric cancer (GC) activates the HGF-c-Met axis, which promotes nuclear translocation of MACC1. This leads to nuclear expression upregulation of TWIST1/2, which facilitates VM by increasing the expression of VEGFR2 and VE-cadherin, decreasing the expression of E-cadherin. Then VM promotes GC cell growth and metastasis. ↑: activation, ┴: inhibition.

In summary, we carried out substantial experiments to determine the mechanism of MACC1-induced VM in GC. We confirmed that MACC1 expression is correlated with VM density, and that positivity for both MACC1 and VM predicts a worse clinical outcome for GC patients. Our findings indicate that MACC1 promotes VM in GC by the signaling pathway displayed in Figure 8. In conclusion, MACC1 promotes VM in GC by regulating the HGF/c-Met-TWIST1/2 signaling pathway, which means that MACC1 and this pathway are potential new therapeutic targets for GC.

## MATERIALS AND METHODS

### Patients and tumor tissue samples

Tumor specimens of 88 patients with stage IV GC were obtained from the Tumor Tissue Bank of Nanfang Hospital (Guangzhou, China). All of the patients were diagnosed with primary GC and underwent palliative surgery or biopsy in Nanfang Hospital from 2005 to 2010. Formalin-fixed tumor tissues were used for immunohistochemistry (IHC), while fresh tumor samples and matching adjacent non-tumor tissues from 5 patients were used for molecular biological analyses. Detailed clinicopathological data were recorded, including each patient's age, gender, tumor differentiation, lymph node metastasis, and survival time. Complete data were available for all patients and the follow-up period ranged from 0.4 to 47.0 months (median: 7.4 months). This study was approved by the ethics committee of Southern Medical University.

### Animal models

Subcutaneous GC implantation and lung metastasis models were created in NOD-SCID nude mice as described previously [14]. OxMACC1 and vector-control cells or shMACC1 and scramble-control GC cells (1 × 10^7^ cells/mouse or 2 × 10^6^ cells/mouse, respectively; *n* = 6) were separately inoculated into the left and right sides of the back or injected into the tail vein. The mice were sacrificed on day 18 or 40, subcutaneous GC tumors and lung metastases were harvested, and examination was performed after H&E or IHC staining.

### Three-dimensional culture

A 24-well culture plate was coated with 250 μl/well of growth factor-reduced Matrigel (BD Biosciences, Franklin Lakes, NJ), which was allowed to polymerize for 1 hour at 37°C. Then a cell suspension (1 × 10^5^ cells/well) was seeded on top of the gel, and three wells were provided for each group. After adding DMEM (Invitrogen, Carlsbad, CA, USA) containing 10% fetal bovine serum (Thermo Scientific HyClone, South Logan, UT, USA), the cells were continuously observed for 12–48 hours in the 3D culture system. Cells were photographed under an inverted microscope (Olympus CKX41; Olympus, Tokyo, Japan). An independent observer counted the total number of tube-like structures per image [2]. Cells were collected from Matrigel by trypsinization (BD Biosciences) and total RNA or total protein was isolated with Trizol (Bio-Rad Lab., Richmond, CA, USA) or RIPA buffer (Beyotime, Shanghai, China), respectively.

### Cell culture, plasmid, and transfection of GC cell lines

Human gastric adenocarcinoma cell lines of poorly differentiated BGC-823 and well-differentiated MKN-28 were obtained from Foleibao Biotechnology Development Co. (Shanghai, China). Poorly differentiated cancer cell lines (human colon cancer cell lines HCT-116 and SW480, breast cancer cell lines MDA-MB-231 and BT549, and lung cancer cell lines A549 and NCIH520) were purchased from American Type Culture Collection (ATCC, Manassas, VA, USA). BGC-823 and MKN-28 cells stably expressing MACC1 cDNA or MACC1-specific shRNA were described in our previous study [14]. Vector and Scramble plasmids were used to the corresponding controls as previously described [14]. All cells were stored at early passages in our laboratory. Cell lines were authenticated by short tandem repeat and genotyped upon re-expansions, and experiments were carried out with low passage cultures of these stocks. The cells were maintained with DMEM (Invitrogen) supplemented with 10% FBS (Thermo Scientific HyClone), 0.5 μg/mL puromycin under conditions of 5% CO_2_ at 37°C as described previously [14]. BGC-823 and MKN-28 cells expressing TWIST1-specific or TWIST2-specific siRNAs (Sigma, St. Louis, MO, USA) were performed by Lipofectamine 2000 (Invitrogen). A nonspecific scrambled siRNA (siCTR) was used as a control to examine the effect of transfection (Table S5). HCT-116, BT549 and NCIH520 cells expressing MACC1-specific shRNA were also performed by Lipofectamine 2000 (Invitrogen). Scramble plasmids were used severed the corresponding controls as previously described [14].

In some experiments, recombinant human hepatocyte growth factor (rhHGF; PeproTech, Rocky Hill, NJ, USA) and the c-Met inhibitor PF-04217903 (Sigma) were used at the indicated concentrations and durations.

### Histological examination

IHC was performed and staining scores were determined as reported elsewhere [14]. The sections were incubated with a series of primary and secondary antibodies (Table S6). Ten fields of each tissue section were randomly selected under the microscope at a magnification of 400 ×, and 100 cells were counted in each field. An IHC score ≥ 3 was defined as positive expression.

Following IHC, the sections were counterstained with hematoxylin and PAS. Ten fields of each section were randomly selected under the microscope at a magnification of 200 ×, and the VM density (the number of VM per mm^2^) was calculated [5, 45]. A VM density ≥ 10.9 was defined as a high density. The results of double-staining were quantified by the method of Bittner et al [46].

### Transmission electron microscopy

Tissue samples were fixed in 2.5% glutaraldehyde and post-fixed in 1% osmium tetroxide, and then were dehydrated and embedded according to the standard procedure. The presence of red blood cells in channels without endothelium was used as the criterion for identifying VM, while red blood cells in channels with endothelium indicated vessels formed by endothelium-dependent angiogenesis. After staining with uranyl acetate and lead citrate, the sections were observed by using a Hitachi H-7500 transmission electron microscope (Hitachi, Tokyo, Japan).

### MTT assay

GC cells were seeded into 96-well plates (8 × 10^3^ cells/well) with 100 μL of cell suspension and incubated for 7 days. The 3-(4, 5 dimethylthiazol-2-yl)-2, 5-diphenyletrazolium bromide (MTT) assay was performed by adding 20 μL of MTT (5 mg/ml; Promega, Madison, WI, USA) and incubating the plates for 4 hours until a purple precipitate was visible. Precipitates were dissolved in 150 μL of dimethyl sulfoxide and the absorbance of each well was measured at 570 nm with a microplate reader. Each experiment was repeated three times.

### Flow cytometric analysis

Cells were harvested in the exponential growth phase and washed twice with phosphate-buffered saline (PBS). Then the cells were fixed and stained with FITC-conjugated antibodies for VE-cadherin, VEGFR2 (Cell Signaling Technology, Beverly, MA, USA), and isotype IgG (Sigma). Flow cytometry was performed with a FAC Scan (BD Biosciences) and the data were analyzed with WinMDI 2.8 software (Verity Software House, San Diego, USA). Plots of side scatter (SSC) versus FITC fluorescence were created in which R2 indicated the gated regions.

### Luciferase reporter assay

The TWIST1 and TWIST2 promoter regions (2kb surrounding the transcription start site) were generated by amplification of genomic DNA (sequences in Table S7) with the polymerase chain reaction (PCR) and were cloned into the pGL3-basic promoter vector (Promega). HEK-293T cells (Guangzhou Institute of Biomedicine and Health, Guangzhou, China) were transfected with TWIST1/2 promoter plasmids or the control plasmid and oxMACC1 plasmid. At 48 hours after transfection, luciferase activity was analyzed by the Dual Luciferase system according to the manufacturer's instructions (Promega). Firefly luciferase activity was normalized to that of Renilla luciferase. Each experiment was repeated three times.

### Western blot analysis

Western blotting was performed as described previously [14], using anti-MACC1 (Abcam, Cambridge, MA, USA), anti-TWIST1 (Santa Cruz Biotechnology, Santa Cruz, CA, USA), anti-TWIST2 (Abcam), anti-VE-cadherin, anti-VEGFR2 and anti-E-cadherin antibodies (Cell Signaling Technology). For subcellular localization of proteins, total cell extracts and nuclear extracts were prepared using an extraction kit (Beyotime). GAPDH (KangCheng, Shanghai, China) and lamin (Cell Signaling Technology) served as the loading controls.

### Quantitative real-time PCR

The sequences of the primers for VE-cadherin, VEGFR2, E-cadherin, and GAPDH are summarized in Table S8. Total RNA was extracted from GC cells using a Trizol kit (Bio-Rad) according to the manufacturer's instructions. Then 1 μg of RNA was reverse transcribed using a First Strand cDNA Synthesis kit (Takara, Carlsbad, CA, USA) and quantitative PCR was performed with SYBR Green dye (Roche Diagnostics, Mannheim, Germany). Relative levels of mRNA expression were analyzed using a LightCycler 480 system (Roche Diagnostics).

### Statistical analysis

Data are presented as the mean ± standard error of the mean (SEM). Statistical analysis was conducted with SPSS 13.0 software (SPSS Inc., Chicago, IL, USA). Student's *t*-test was used to compare continuous variables between two groups, while the chi-square test was applied for comparison of dichotomous variables. Correlations were assessed by the nonparametric Spearman-Rho method. The Kaplan-Meier method was used for survival analysis, and differences of survival between groups were assessed with the log-rank test. Multivariate analysis was performed with a Cox model. In all analyses, significance was accepted at *p* < 0.05.

## SUPPLEMENTAL FIGURES AND TABLES



## References

[1] Hanahan D, Folkman J (1996). Patterns and emerging mechanisms of the angiogenic switch during tumorigenesis. Cell.

[2] Maniotis AJ, Folberg R, Hess A, Seftor EA, Gardner LM, Pe'er J, Trent JM, Meltzer PS, Hendrix MJ (1999). Vascular channel formation by human melanoma cells *in vivo* and *in vitro*: vasculogenic mimicry. Am J Pathol.

[3] Zhang S, Guo H, Zhang D, Zhang W, Zhao X, Ren Z, Sun B (2006). Microcirculation patterns in different stages of melanoma growth. Oncol Rep.

[4] Liu T, Sun B, Zhao X, Gu Q, Dong X, Yao Z, Zhao N, Chi J, Liu N, Sun R, Ma Y (2013). HER2/neu expression correlates with vasculogenic mimicry in invasive breast carcinoma. J Cell Mol Med.

[5] Li M, Gu Y, Zhang Z, Zhang S, Zhang D, Saleem AF, Zhao X, Sun B (2010). Vasculogenic mimicry: a new prognostic sign of gastric adenocarcinoma. Pathol Oncol Res.

[6] Liu WB, Xu GL, Jia WD, Li JS, Ma JL, Chen K, Wang ZH, Ge YS, Ren WH, Yu JH, Wang W, Wang XJ (2011). Prognostic significance and mechanisms of patterned matrix vasculogenic mimicry in hepatocellular carcinoma. Med Oncol.

[7] Xu Y, Li Q, Li XY, Yang QY, Xu WW, Liu GL (2012). Short-term anti-vascular endothelial growth factor treatment elicits vasculogenic mimicry formation of tumors to accelerate metastasis. J Exp Clin Cancer Res.

[8] Wilke H, Muro K, Van Cutsem E, Oh SC, Bodoky G, Shimada Y, Hironaka S, Sugimoto N, Lipatov O, Kim TY, Cunningham D, Rougier P, Komatsu Y, Ajani J, Emig M, Carlesi R (2014). Ramucirumab plus paclitaxel versus placebo plus paclitaxel in patients with previously treated advanced gastric or gastro-oesophageal junction adenocarcinoma (RAINBOW): a double-blind, randomised phase 3 trial. The Lancet Oncology.

[9] Allegra CJ, Yothers G, O'Connell MJ, Sharif S, Petrelli NJ, Colangelo LH, Atkins JN, Seay TE, Fehrenbacher L, Goldberg RM, O'Reilly S, Chu L, Azar CA, Lopa S, Wolmark N (2011). Phase III trial assessing bevacizumab in stages II and III carcinoma of the colon: results of NSABP protocol C-08. J Clin Oncol.

[10] McLemore MR (2006). The role of the data safety monitoring board: why was the Avastin phase III clinical trial stopped?. Clin J Oncol Nurs.

[11] Wang JY, Sun T, Zhao XL, Zhang SW, Zhang DF, Gu Q, Wang XH, Zhao N, Qie S, Sun BC (2008). Functional significance of VEGF-a in human ovarian carcinoma - Role in vasculogenic mimicry. Cancer Biology & Therapy.

[12] El Hallani S, Boisselier B, Peglion F, Rousseau A, Colin C, Idbaih A, Marie Y, Mokhtari K, Thomas JL, Eichmann A, Delattre JY, Maniotis AJ, Sanson M (2010). A new alternative mechanism in glioblastoma vascularization: tubular vasculogenic mimicry. Brain : a journal of neurology.

[13] Jiang J, Liu W, Guo X, Zhang R, Zhi Q, Ji J, Zhang J, Chen X, Li J, Zhang J, Gu Q, Liu B, Zhu Z, Yu Y (2011). IRX1 influences peritoneal spreading and metastasis via inhibiting BDKRB2-dependent neovascularization on gastric cancer. Oncogene.

[14] Wang L, Wu Y, Lin L, Liu P, Huang H, Liao W, Zheng D, Zuo Q, Sun L, Huang N, Shi M, Liao Y, Liao W (2013). Metastasis-associated in colon cancer-1 upregulation predicts a poor prognosis of gastric cancer, and promotes tumor cell proliferation and invasion. Int J Cancer.

[15] Stein U, Walther W, Arlt F, Schwabe H, Smith J, Fichtner I, Birchmeier W, Schlag PM (2009). MACC1, a newly identified key regulator of HGF-MET signaling, predicts colon cancer metastasis. Nat Med.

[16] Sanchez-Tillo E, Liu Y, de Barrios O, Siles L, Fanlo L, Cuatrecasas M, Darling DS, Dean DC, Castells A, Postigo A (2012). EMT-activating transcription factors in cancer: beyond EMT and tumor invasiveness. Cell Mol Life Sci.

[17] Thiery JP, Acloque H, Huang RY, Nieto MA (2009). Epithelial-mesenchymal transitions in development and disease. Cell.

[18] Sun T, Sun BC, Zhao XL, Zhao N, Dong XY, Che N, Yao Z, Ma YM, Gu Q, Zong WK, Liu ZY (2011). Promotion of tumor cell metastasis and vasculogenic mimicry by way of transcription coactivation by Bcl-2 and Twist1: a study of hepatocellular carcinoma. Hepatology.

[19] Liu Z, Sun B, Qi L, Li H, Gao J, Leng X (2012). Zinc finger E-box binding homeobox 1 promotes vasculogenic mimicry in colorectal cancer through induction of epithelial-to-mesenchymal transition. Cancer Sci.

[20] Yang J, Mani SA, Donaher JL, Ramaswamy S, Itzykson RA, Come C, Savagner P, Gitelman I, Richardson A, Weinberg RA (2004). Twist, a master regulator of morphogenesis, plays an essential role in tumor metastasis. Cell.

[21] Sung CO, Lee KW, Han S, Kim SH (2011). Twist1 is up-regulated in gastric cancer-associated fibroblasts with poor clinical outcomes. Am J Pathol.

[22] Eckert MA, Lwin TM, Chang AT, Kim J, Danis E, Ohno-Machado L, Yang J (2011). Twist1-induced invadopodia formation promotes tumor metastasis. Cancer Cell.

[23] Sun T, Zhao N, Zhao XL, Gu Q, Zhang SW, Che N, Wang XH, Du J, Liu YX, Sun BC (2010). Expression and Functional Significance of Twist1 in Hepatocellular Carcinoma: Its Role in Vasculogenic Mimicry. Hepatology.

[24] Franco HL, Casasnovas J, Rodriguez-Medina JR, Cadilla CL (2011). Redundant or separate entities?-roles of Twist1 and Twist2 as molecular switches during gene transcription. Nucleic Acids Research.

[25] Ansieau S, Bastid J, Doreau A, Morel AP, Bouchet BP, Thomas C, Fauvet F, Puisieux I, Doglioni C, Piccinin S, Maestro R, Voeltzel T, Selmi A, Valsesia-Wittmann S, Caron de Fromentel C, Puisieux A (2008). Induction of EMT by twist proteins as a collateral effect of tumor-promoting inactivation of premature senescence. Cancer Cell.

[26] Li Y, Wang W, Wang W, Yang R, Wang T, Su T, Weng D, Tao T, Li W, Ma D, Wang S (2012). Correlation of TWIST2 up-regulation and epithelial-mesenchymal transition during tumorigenesis and progression of cervical carcinoma. Gynecol Oncol.

[27] Comito G, Calvani M, Giannoni E, Bianchini F, Calorini L, Torre E, Migliore C, Giordano S, Chiarugi P (2011). HIF-1alpha stabilization by mitochondrial ROS promotes Met-dependent invasive growth and vasculogenic mimicry in melanoma cells. Free Radic Biol Med.

[28] Hendrix MJ, Seftor EA, Meltzer PS, Gardner LM, Hess AR, Kirschmann DA, Schatteman GC, Seftor RE (2001). Expression and functional significance of VE-cadherin in aggressive human melanoma cells: role in vasculogenic mimicry. Proc Natl Acad Sci U S A.

[29] Ferrara N, Gerber HP, LeCouter J (2003). The biology of VEGF and its receptors. Nat Med.

[30] Ebos JM, Lee CR, Cruz-Munoz W, Bjarnason GA, Christensen JG, Kerbel RS (2009). Accelerated metastasis after short-term treatment with a potent inhibitor of tumor angiogenesis. Cancer Cell.

[31] Cao Z, Bao M, Miele L, Sarkar FH, Wang Z, Zhou Q (2013). Tumour vasculogenic mimicry is associated with poor prognosis of human cancer patients: a systemic review and meta-analysis. European journal of cancer (Oxford, England: 1990).

[32] Petty AP, Garman KL, Winn VD, Spidel CM, Lindsey JS (2007). Overexpression of carcinoma and embryonic cytotrophoblast cell-specific Mig-7 induces invasion and vessel-like structure formation. Am J Pathol.

[33] Rahman MA, Salajegheh A, Smith RA, Lam AK (2013). B-Raf mutation: a key player in molecular biology of cancer. Experimental and molecular pathology.

[34] Takashima A, Faller DV (2013). Targeting the RAS oncogene. Expert opinion on therapeutic targets.

[35] Linardou H, Dahabreh IJ, Kanaloupiti D, Siannis F, Bafaloukos D, Kosmidis P, Papadimitriou CA, Murray S (2008). Assessment of somatic k-RAS mutations as a mechanism associated with resistance to EGFR-targeted agents: a systematic review and meta-analysis of studies in advanced non-small-cell lung cancer and metastatic colorectal cancer. The Lancet Oncology.

[36] Bergers G, Hanahan D (2008). Modes of resistance to anti-angiogenic therapy. Nature reviews Cancer.

[37] Ricci-Vitiani L, Pallini R, Biffoni M, Todaro M, Invernici G, Cenci T, Maira G, Parati EA, Stassi G, Larocca LM, De Maria R (2010). Tumour vascularization via endothelial differentiation of glioblastoma stem-like cells. Nature.

[38] Merindol N, Riquet A, Szablewski V, Eliaou JF, Puisieux A, Bonnefoy N (2014). The emerging role of Twist proteins in hematopoietic cells and hematological malignancies. Blood cancer journal.

[39] Valsesia-Wittmann S, Magdeleine M, Dupasquier S, Garin E, Jallas AC, Combaret V, Krause A, Leissner P, Puisieux A (2004). Oncogenic cooperation between H-Twist and N-Myc overrides failsafe programs in cancer cells. Cancer Cell.

[40] Stein U, Smith J, Walther W, Arlt F (2009). MACC1 controls Met What a difference an Sp1 site makes. Cell Cycle.

[41] Juneja M, Ilm K, Schlag PM, Stein U (2013). Promoter identification and transcriptional regulation of the metastasis gene MACC1 in colorectal cancer. Molecular Oncology.

[42] Entz-Werle N, Lavaux T, Metzger N, Stoetzel C, Lasthaus C, Marec P, Kalifa C, Brugieres L, Pacquement H, Schmitt C, Tabone MD, Gentet JC, Lutz P, Babin A, Oudet P, Gaub MP (2007). Involvement of MET/TWIST/APC combination or the potential role of ossification factors in pediatric high-grade osteosarcoma oncogenesis. Neoplasia.

[43] Mani SA, Guo W, Liao MJ, Eaton EN, Ayyanan A, Zhou AY, Brooks M, Reinhard F, Zhang CC, Shipitsin M, Campbell LL, Polyak K, Brisken C, Yang J, Weinberg RA (2008). The epithelial-mesenchymal transition generates cells with properties of stem cells. Cell.

[44] Shimada S, Mimata A, Sekine M, Mogushi K, Akiyama Y, Fukamachi H, Jonkers J, Tanaka H, Eishi Y, Yuasa Y (2012). Synergistic tumour suppressor activity of E-cadherin and p53 in a conditional mouse model for metastatic diffuse-type gastric cancer. Gut.

[45] Vartanian A, Stepanova E, Grigorieva I, Solomko E, Baryshnikov A, Lichinitser M (2011). VEGFR1 and PKCalpha signaling control melanoma vasculogenic mimicry in a VEGFR2 kinase-independent manner. Melanoma research.

[46] Bittner M, Meitzer P, Chen Y, Jiang Y, Seftor E, Hendrix M, Radmacher M, Simon R, Yakhini Z, Ben-Dor A, Sampas N, Dougherty E, Wang E, Marincola F, Gooden C, Lueders J (2000). Molecular classification of cutaneous malignant melanoma by gene expression profiling. Nature.

