# Supplementary material for: Metastasis-associated in colon cancer-1 promotes vasculogenic mimicry in gastric cancer by upregulating TWIST1/2
Source: Oncotarget. 2015 Mar 20;6(13):11492–506. doi: 10.18632/oncotarget.3416 (PMC4484471; doi:10.18632/oncotarget.3416)
Supplement: Supplementary file 1 [file oncotarget-06-11492-s001.pdf]

SUPPLEMENTAL FIGURES AND TABLES

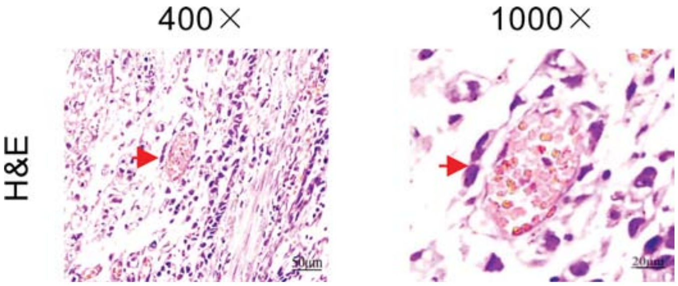

Supplementary Figure S1: Representative pictures of vasculogenic mimicry (VM) structure confirmed by H&E staining. Several red blood cells (red arrows) are surrounded by tumor cells.

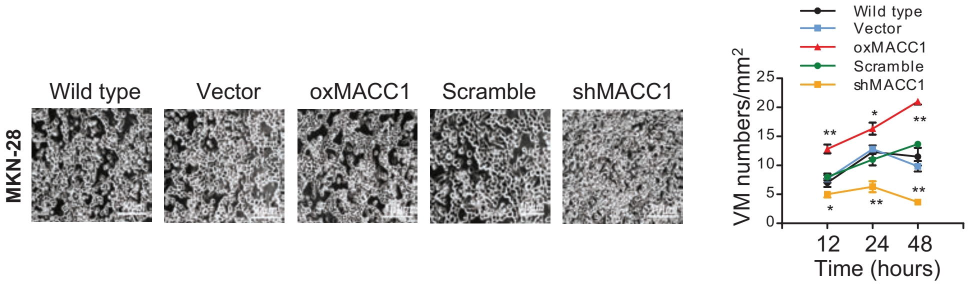

Supplementary Figure S2: MACC1 promoted VM formation in MKN-28 GC cell line. Representative VM images and quantitation of tube formation by MKN-28 GC cells cultured in 3D system for 48 hours. Scale bar = 50 µm. $*p < 0.05$; $**p < 0.01$, $n = 3$ in each group.

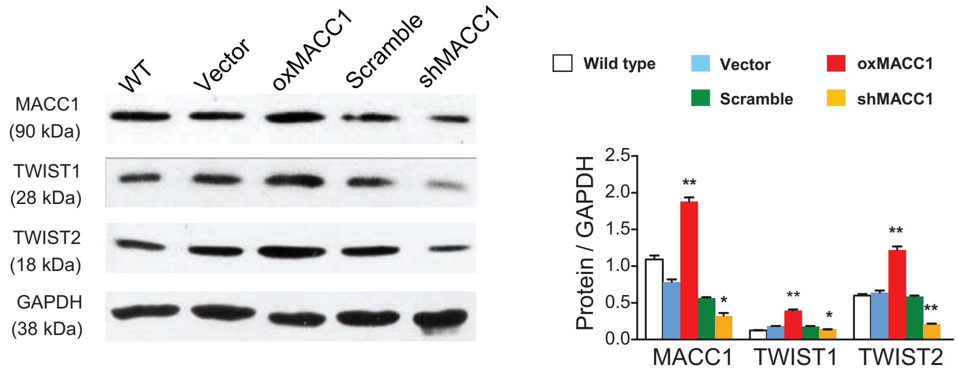

Supplementary Figure S3: MACC1 upregulated TWIST1 and TWIST2 expression in MKN28 GC cell line. Western blot analysis and quantitation of MACC1, TWIST1 and TWIST2 expression in response to overexpressing or silencing of MACC1 (oxMACC1 and shMACC1) in MKN-28 GC cell line. *$p < 0.05$; **$p < 0.01$, $n = 3$. WT, wild type. GAPDH was used as a loading control.

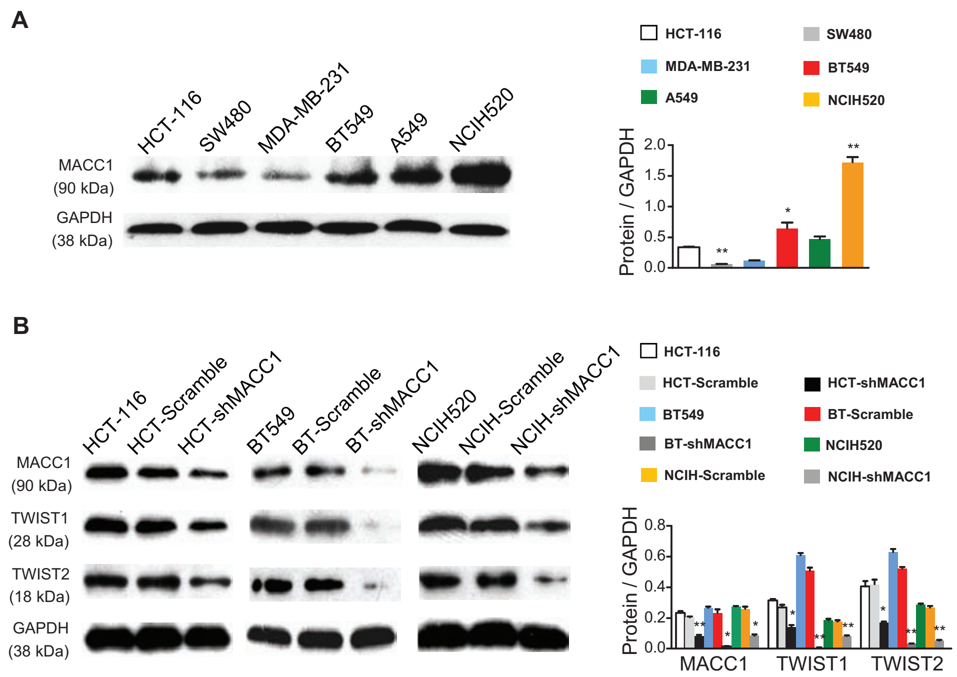

Supplementary Figure S4: Silencing MACC1 downregulated TWIST1 and TWIST2 expression in colon cancer, breast cancer and lung cancer cell lines. A. Western blot analysis and quantitation of MACC1 expression in colon cancer (HCT-116 and SW480), breast cancer (MDA-MB-231 and BT549) and lung cancer (A549 and NCIH520) cell lines. **B.** MACC1, TWIST1 and TWIST2 expressions were downregulated in response to silencing of MACC1 (shMACC1) in colon cancer, breast cancer and lung cancer cell lines. GAPDH was used as a loading control. **p* < 0.05; ***p* < 0.01, *n* = 3.

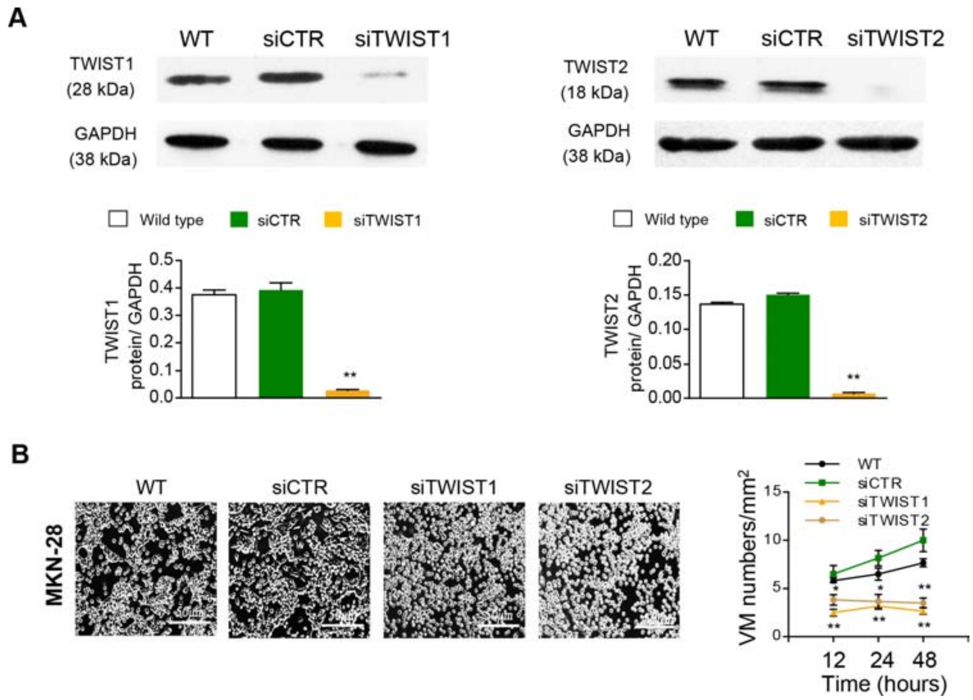

Supplementary Figure S5: Silencing TWIST1/2 attenuated VM formation in MKN-28 GC cell line. A. TWIST1 and TWIST2 proteins were silenced by corresponding siRNA sequences. **B.** Representative images of VM density (tube formation) at 48 hours of treatment and the time course quantitation of VM density in 3D cultures of MKN-28 cells treated with silenced TWIST1, TWIST2 or the siCTR control. $*p < 0.05$; $**p < 0.01$, $n = 3$ in each group.

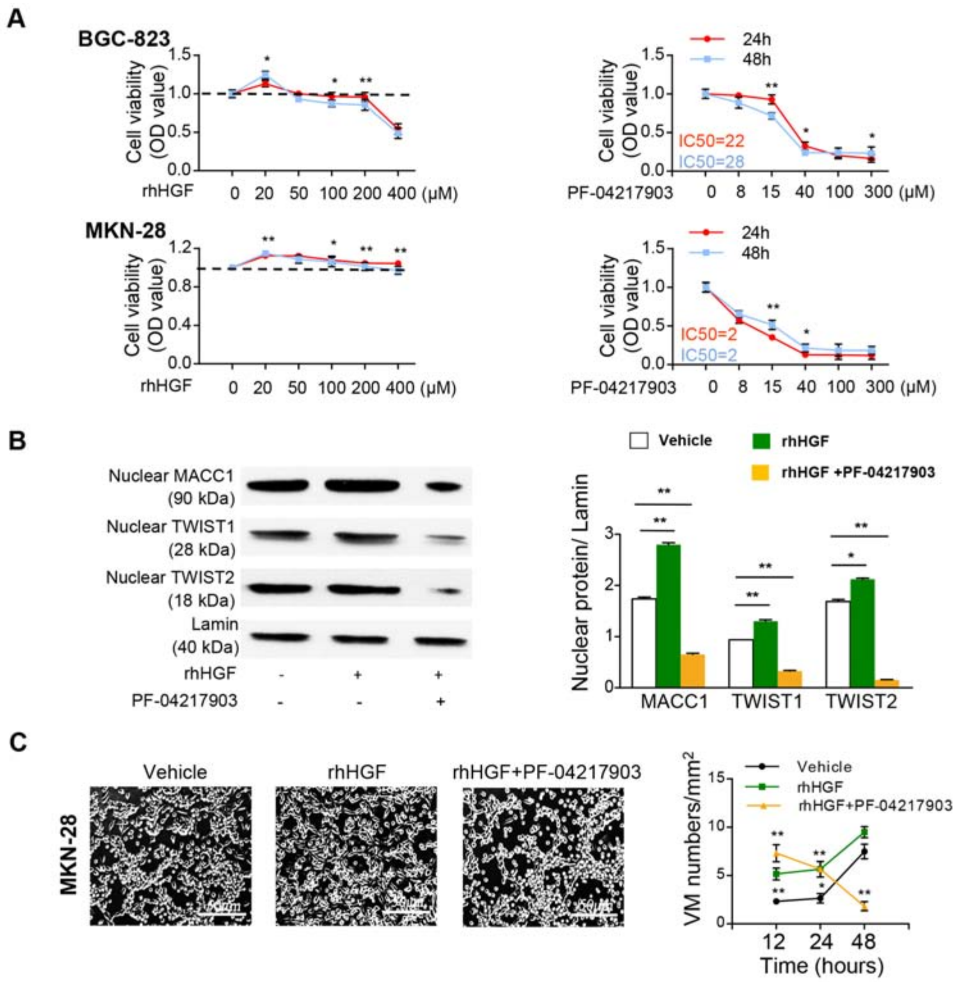

Supplementary Figure S6: HGF/c-Met contributed to MACC1-induced VM formation in MKN-28 GC cell line. A. MTT assays showed the maximal proliferation concentration of recombinant human hepatocyte growth factor (rhHGF) and the half maximal inhibitory concentration (IC50) of c-Met inhibitor PF-04217903 in BGC-823 and MKN-28 GC cell lines at 24 h and 48 h. **B.** Nuclear expression of TWIST1 and TWIST2 were upregulated by rhHGF, which were antagonized by co-treatment with PF-04217903. **C.** Representative pictures and time course effect of rhHGF, rhHGF+PF-04217903 or vehicle on the density of tube formation were taken 48 hours. Scale bar = 50 μm. $*p < 0.05$; $**p < 0.01$, $n = 3$. The concentrations for rhHGF and PF-04217903 were 20 μM and 2 μM, respectively.

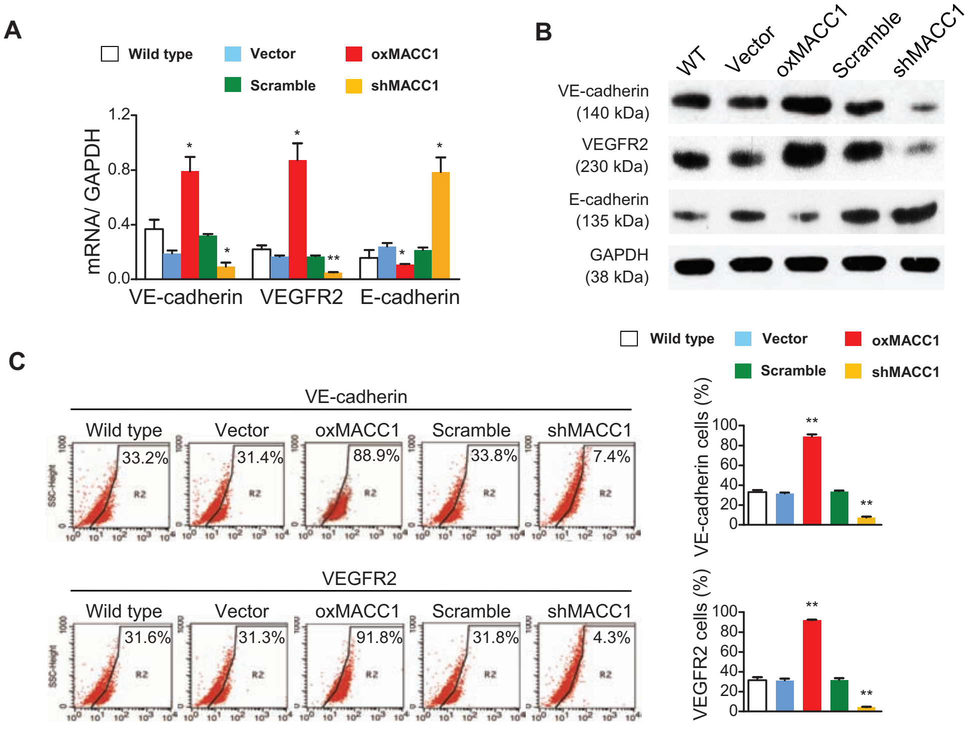

Supplementary Figure S7: Effect of MACC1 on expression of VE-cadherin, VEGFR2 and E-cadherin. MACC1-overexpressing (oxMACC1) or MACC1-silencing (shMACC1) changed the mRNA **A.** and protein expression **B.** of VE-cadherin, VEGFR2 and E-cadherin in MKN-28 cells. **C.** VE-cadherin and VEGFR2 expressions in MKN-28 cells were measured by flow cytometry. $*p < 0.05$; $**p < 0.01$, $n = 3$ in each group.

Supplementary Table S1. Correlation between VM and Clinicopathologic characteristics in 88 stage IV GC tissues

Characteristic	*n* (%)	VM density		χ^2	*P* value
		High n (%)	Low n (%)		
**Survival in stage IV gastric cancer (GC)**					
		38 (43.2%)	50 (56.8%)		
Gender				0.452	0.501
Male	52 (59.1%)	24 (46.2%)	28 (53.8%)		
Female	36 (40.9%)	14 (38.9%)	22 (61.1%)		
Age (years)				0.551	0.458
≥ 55	40 (45.5%)	19 (47.5%)	21 (52.5%)		
< 55	48 (54.5%)	19 (39.6%)	29 (60.4%)		
Differentiation				5.602	**0.018**
WD/MD	14 (15.9%)	2 (14.3%)	12 (85.7%)		
PD	74 (84.1%)	36 (48.6%)	38 (51.4%)		
Depth of invasion				0.074	0.786
T1/T2/T3	15 (17.0%)	6 (40.0%)	9 (60.0%)		
T4	73 (83.0%)	32 (43.8%)	41 (56.2%)		
Lymph node metastasis				0.114	0.735
N0/N1/N2	8 (9.1%)	3 (37.5%)	5 (62.5%)		
N3	80 (90.9%)	35 (43.7%)	45 (56.3%)		
Metastasis lesions				5.029	0.**025**
< 3	18 (20.5%)	12 (66.7%)	6 (6.8%)		
≥ 3	70 (79.5%)	26 (37.1%)	44 (62.9%)		

Abbreviations: WD, well differentiated; MD, moderately differentiated; PD, poorly differentiated

Supplementary Table S2. Multivariate analysis of various prognosis parameters in 88 GC patients using Cox regression model

Covariates	*P* value	HR (Hazard ratio)	95% CI for Exp (B)	
			Lower	Upper
Survival in stage IV GC				
Gender (vs. Female)	0.341	0.758	0.429	1.340
Age (vs. < 55 years)	0.814	0.480	0.357	1.378
Differentiation (vs. PD)	0.628	1.185	0.596	2.357
Depth of invasion (vs.T4)	0.769	0.906	0.470	1.746
Lymph node metastasis (vs. N3)	0.535	0.759	0.318	1.814
Metastasis lesions (vs. < 3)	**0.009**	2.362	1.240	4.500
VM density (vs. Low)	**0.005**	2.371	1.290	4.359
MACC1 expression (vs. Negative)	**< 0.001**	4.197	1.886	9.339

Supplementary Table S3. Pearson correlation analysis between MACC1 and VM, VE-cadherin expression

Variant	n (%)	MACC1 expression		Pearson Correlation	*P* value
		Positive n (%)	Negative n (%)		
VM density				0.212	**0.047**
High	38 (43.2%)	35 (92.1%)	3 (7.9%)		
Low	50 (56.8%)	38 (76.0%)	12 (24.0%)		
VE-cadherin				0.487	**< 0.001**
Positive	65 (73.9%)	61 (93.8%)	4 (6.2%)		
Negative	23 (26.1%)	12 (52.2%)	11 (47.8%)		

Supplementary Table S4. Pearson correlation analysis between VM and VE-cadherin expression

Variant	n (%)	VM density		Pearson Correlation	*P* value
		High n (%)	Low n (%)		
VE-cadherin				0.258	**0.015**
Positive	65 (73.9%)	33 (50.8%)	32 (49.2%)		
Negative	23 (26.1%)	5 (21.7%)	18 (78.3%)		

Supplementary Table S5. Interference sequences for TWIST1 and TWIST2 silence

Gene name	Primer sequences (5′ to 3′)
SiTWIST1	F-CUCACGAGCGGCUCAGCUAdTdT
	R-UAGCUGACCCGCUCGUGAGdTdT
SiTWIST2	F-CCUUCUCCGUGUGGCGCAUdTdT
	R-AUGCGCCACACGGAGAAGGdTdT
SiCTR	F-UUCUCCGAACGUGUCACGUTT
	R-ACGUGACACGUUCGGAGAATT

Supplementary Table S6. List of proteins tested by antibodies and features of the corresponding antibodies

Protein	Aaasay	Company	Product number	Origin	Dilution	Incubation
MACC1	WB	Abcam	AB-106579	rpab	1/800	overnight, 4°C
MACC1	IHC	Abnova	PAB16755	rpab	1/1000	overnight, 4°C
CD31	IHC	Abcam	AB-28364	rpab	1/50	overnight, 4°C
CK19	IHC	Proteintech	10712–1-AP	rpab	1/800	overnight, 4°C
TWIST1	WB	Santa Cruz	SC-15393	rpab	1/800	overnight, 4°C
TWIST1	IHC	Santa Cruz	SC-15393	rpab	1/50	overnight, 4°C
TWIST2	WB	Abcam	AB-66031	rpab	1/1000	overnight, 4°C
TWIST2	IHC	Abcam	AB-66031	rpab	1/50	overnight, 4°C
VE-cadherin	WB	CST	#2500	rpab	1/1000	overnight, 4°C
VE-cadherin	FC	CST	#2500	rpab	1/100	overnight, 4°C
VE-cadherin	IHC	CST	#2500	rpab	1/500	overnight, 4°C
VEGFR2	WB	CST	#9698	rpab	1/1000	overnight, 4°C
VEGFR2	FC	CST	#9698	rpab	1/400	overnight, 4°C
E-cadherin	WB	CST	#3195	rpab	1/1000	overnight, 4°C

Abbreviations: CST, cell signaling technology; WB, western blot; IHC, immunohistochemistry; FC, flow cytometry; rpab, rabbit polyclonal antibody

Supplementary Table S7. Primer sequences for TWIST1 and TWIST2 promoter

Promoter name	Primer sequences (5′ to 3′)
TWIST1- Luc	F-CGGGGTACCCGTGCAGGCGGAAAGTTTGG
	R-CCCAAGCTTCAGAATACTGTAAATTCAGATTTACAAAAAGAAC
TWIST2- Luc	F-CGACGCGTAAAGAGACAGAAAGGACAGAGAAAGAG
	R-CCGCTCGAGCTGGGCTGGGTTGCTAAATAGTTG

Supplementary Table S8. Primer sequences for quantitative real-time PCR

Gene name	Primer sequences (5′ to 3′)	Primer length (bps)	Amplified fragment length (bps)
VE-cadherin	F-CCTGACTGTGGAGGCCAAAGA	21	144
	R-TTCTCACACACTTTGGGCTGGTAG	24	
VEGFR2	F-CCCTGGAGACCTGAGAACCA	20	180
	R-CCCGAGTGTAACCATAGCGG	20	
E-cadherin	F-GAGTGCCAACTGGACCATTCAGTA	24	86
	R-AGTCACCCACCTCTAAGGCCATC	23	
GAPDH	F-ACTTCAACAGCGACACCCACTC	22	132
	R-TACCAGGAAATGAGCTTGACAAAG	24	

Abbreviations: PCR, polymerase chain reaction
